# SWI/SNF aberrations sensitize pancreatic cancer cells to DNA crosslinking agents

**DOI:** 10.18632/oncotarget.20033

**Published:** 2017-08-08

**Authors:** Jean Davidson, Zhewei Shen, Xue Gong, Jonathan R. Pollack

**Affiliations:** ^1^ Department of Pathology, Stanford University School of Medicine, Stanford, California, USA; ^2^ Department of Urology, Stanford University School of Medicine, Stanford, California, USA; ^3^ Current address: Department of Cardiovascular Research, Stanford University School of Medicine, Stanford, California, USA

**Keywords:** SWI/SNF, pancreatic cancer, DNA crosslinking agents, predictive biomarkers, personalized medicine

## Abstract

While gemcitabine has been the mainstay therapy for advanced pancreatic cancer, newer combination regimens (e.g. FOLFIRINOX) have extended patient survival, though carry greater toxicity. Biomarkers are needed to better stratify patients for appropriate therapy. Previously, we reported that one-third of pancreatic cancers harbor deletions or deleterious mutations in key subunits of the SWItch/Sucrose NonFermentable (SWI/SNF) chromatin remodeling complex. The SWI/SNF complex mobilizes nucleosomes on DNA, and plays a key role in modulating DNA transcription and repair. Thus, we hypothesized that pancreatic cancers with SWI/SNF aberrations might exhibit compromised DNA repair, and show increased sensitivity to DNA damaging agents. Here, we studied human pancreatic cancer cell lines with deficient (or else exogenously reconstituted) SWI/SNF subunits, as well as normal pancreatic epithelial cells following SWI/SNF subunit knockdown. Cells were challenged with DNA damaging agents, including those used in current combination regimens, and then cell viability assayed. We found that pancreatic cells with SWI/SNF dysfunction showed markedly increased sensitivity to DNA damaging agents, and in particular DNA crosslinking agents (cisplatin and oxaliplatin). Assaying clearance of γH2AX confirmed that SWI/SNF dysfunction impaired DNA damage response/repair. Finally, by analyzing pancreatic cancer patient data from The Cancer Genome Atlas, we found that pancreatic cancers with SWI/SNF deficiency (subunit mutation and/or decreased expression) were associated with extended patient survival specifically when treated with platinum containing regimens. Thus, SWI/SNF dysfunction sensitizes pancreatic cancer cells to DNA crosslinking agents, and SWI/SNF mutation status may provide a useful biomarker to predict which patients are likely to benefit from platinum-containing chemotherapy regimens.

## INTRODUCTION

Pancreatic ductal adenocarcinoma (hereafter, pancreatic cancer) carries among the worst prognoses of all human cancers, with a 5-year survival rate of about 8% [1]. Localized pancreatic cancers are treated by surgical excision and adjuvant chemotherapy, while advanced and metastatic cancers are managed by chemotherapy alone [2]. For many years, gemcitabine (a nucleoside analog) has been the mainstay first-line chemotherapy, having shown modest survival benefits over fluorouracil in advanced cancers [3]. More recently, platinum-containing combination chemotherapies – most notably FOLFIRINOX (folinic acid, fluorouracil, irinotecan, and oxaliplatin) – have been found to be superior to gemcitabine [4]. However, such regimens are also more toxic and associated with more side effects, and thus not tolerated by many patients, particularly those older, sicker or with comorbidities. It is unknown whether some patients’ tumors might respond better to combination chemotherapy regimens, and therefore worth the tradeoff of higher toxicity. There are currently no tumor biomarkers to predict therapy response.

Pancreatic cancer development is driven by somatic mutations in well-known cancer genes, including frequent activating mutations of the *KRAS* oncogene, and inactivating mutations of the *CDKN2A* (p16^INK4A^), *SMAD4*, and *TP53* tumor suppressor genes [5]. However, knowledge of these mutations has yet to be translated to improved disease management and patient survival. Thus, new insight is needed, and indeed major efforts have been undertaken to comprehensively catalog the full spectrum of disease-causing alterations in pancreatic cancer [6].

Recently, by array- and sequencing-based profiling of pancreatic cancer genomes, we uncovered deleterious deletions and mutations targeting key subunits of the SWItch/Sucrose Non-Fermentable (SWI/SNF) chromatin remodeling complex in at least one-third of pancreatic cancers [7], findings since confirmed by others [6, 8]. SWI/SNF (also referred to as BAF and PBAF complex) is a multi-subunit complex, conserved from yeast to humans, that uses the energy of ATP hydrolysis to reposition nucleosomes, and thus control the access of transcription factors to DNA [9–11]. Human SWI/SNF complexes contain either of two alternative catalytic (ATPase) subunits, SMARCA4 (BRG1) or SMARCA2 (BRM), one of three alternative DNA targeting/specificity subunits, ARID1A, ARID1B or PBRM1, as well as 8–10 other subunits.

SWI/SNF mutations have now been observed across many cancer types, and collectively in about 20% of all human cancers [12, 13]. In some cancer types, mutations are found predominantly in one specific subunit, e.g. *ARID1A* mutations in ovarian clear cell carcinoma [14], suggesting likely tissue and tumor-type specific functionality of SWI/SNF complexes. How SWI/SNF dysfunction contributes to cancer remains incompletely understood, though SWI/SNF has been reported to regulate the expression or activity of several cancer-relevant genes and pathways (in a variety of tumor types and model systems), including MYC, the RB pathway, the Hedgehog pathway, and Polycomb Repressive complexes [10]. Based in part on that knowledge, therapeutic strategies have begun emerging to specifically target cancers with SWI/SNF aberrations [15].

While most interest has focused on the role of SWI/SNF in regulating gene expression, SWI/SNF is also critical for DNA damage repair [16], including damage caused by ultraviolet light and DNA crosslinking agents [17–19]. In particular, SWI/SNF has been shown to promote the phosphorylation and recruitment of the modified histone H2AX to DNA double-strand breaks, an early step in DNA double-strand break repair [20]. Further, in head and neck squamous cell carcinoma, non-small cell lung cancer, and osteosarcoma cell lines, SWI/SNF dysfunction has been found to increase cellular sensitivity to DNA damaging agents, including cisplatin [18, 19]. Thus, we have hypothesized that the SWI/SNF aberrations commonly observed in pancreatic cancers might sensitize those tumors to DNA damaging agents. Here, we sought to test whether knockdown or re-expression of key SWI/SNF subunits might alter the sensitivity of pancreatic epithelial cells to DNA damaging agents, and if so whether the presence of SWI/SNF aberrations in patient tumors might correlate with responsiveness to DNA-damaging chemotherapy regimens.

## RESULTS

### SMARCA4 knockdown sensitizes pancreatic ductal epithelial cells to DNA damaging agents

SWI/SNF complexes not only function in regulating transcription, but also play important roles in DNA damage repair [16]. Thus, we have postulated that SWI/SNF dysfunction might sensitize pancreatic cancer cells to DNA damaging agents. To investigate, we first modeled SWI/SNF dysfunction in normal (HPV16 E6/E7-immortalized) human pancreatic ductal epithelial (HPDE) cells [21], by RNA interference (RNAi) mediated knockdown of the SWI/SNF catalytic subunit SMARCA4 (BRG1). We then challenged the SMARCA4-knockdown cells to a variety of chemotherapeutic agents, focusing on those that have been evaluated clinically in the treatment of pancreatic cancer [22].

**Figure 1:**
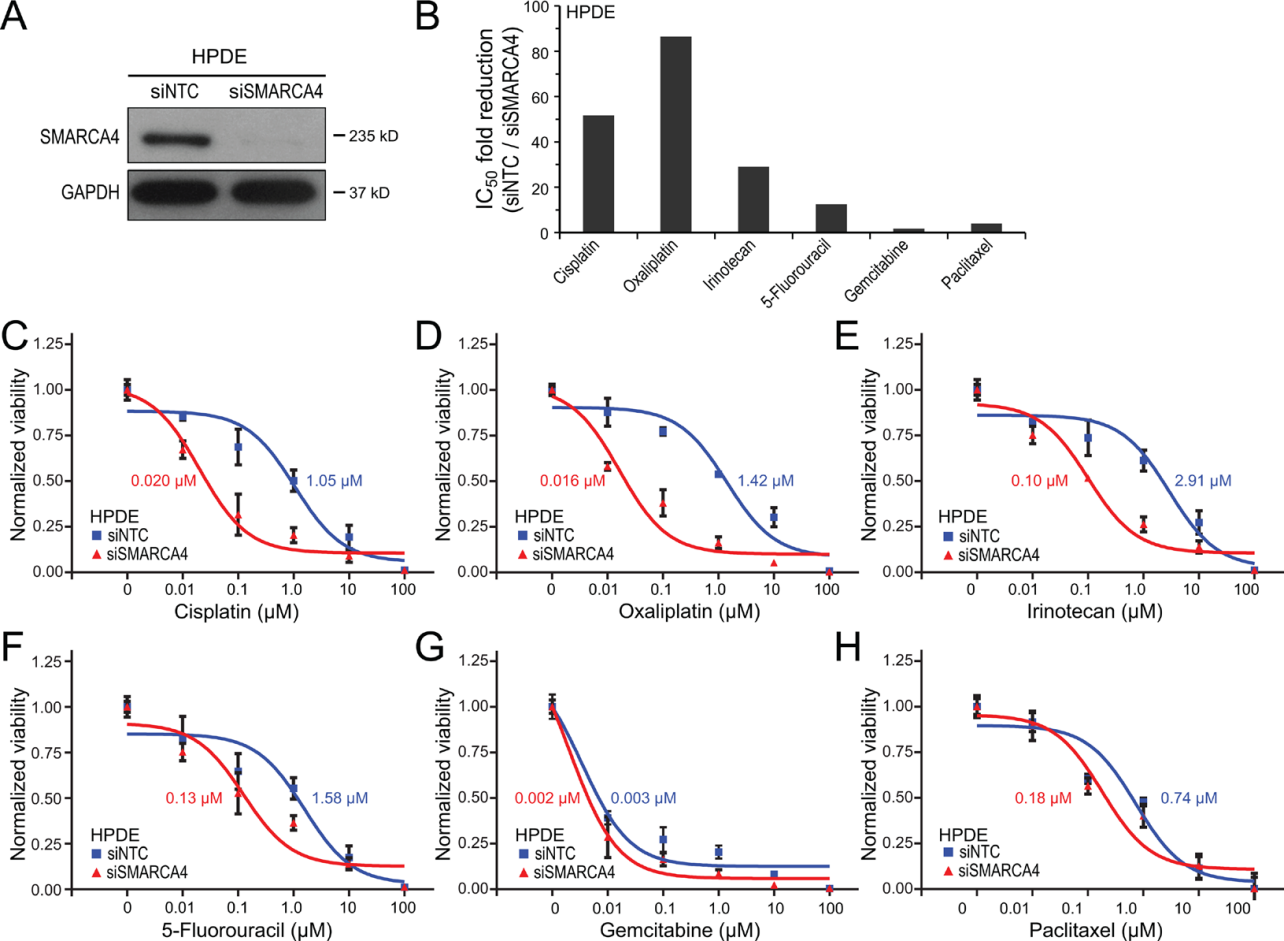
SMARCA4 knockdown sensitizes human pancreatic ductal epithelial cells to DNA-damaging agents (**A**) SMARCA4 knockdown in HPDE cells by siRNA, confirmed by western blot, compared to non-targeting control (NTC); GAPDH serves as a loading control. (**B**) Bar graph summarizing fold-reductions in IC_50_ with SMARCA4 (*vs.* NTC) knockdown, for 6 different chemotherapy agents. (C-H) Dose-response curves comparing HPDE cell viability with SMARCA4 (*vs.* NTC) knockdown, for (**C**) cisplatin, (**D**) oxaliplatin, (**E**) irinotecan, (**F**) 5-fluoruracil, (**G**) gemcitabine, and (**H**) paclitaxel. IC_50_ values indicated.

SMARCA4 knockdown, utilizing an ON-TARGETplus siRNA pool, effectively reduced SMARCA4 protein levels by greater than 95% in HPDE cells, as verified by western blot (Figure 1A). We first challenged the HPDE cells with SMARCA4 knockdown (in comparison to non-targeting siRNA control cells) to a six-point 10-fold serial dilution series of cisplatin, a DNA crosslinking agent that induces DNA double-strand breaks, and then assayed cell viability (by WST-1 assay) at 72 hrs. Notably, SMARCA4-knockdown led to a substantial, 52-fold increased sensitivity to cisplatin, determined by a reduction in the IC_50_ value (50% inhibitory concentration) to 0.02 µM, compared to 1.05 µM in the non-targeting control cells (Figure 1B, 1C). We observed a similar result using oxaliplatin, another platinum-based DNA crosslinking agent currently used against pancreatic cancer in combinations such as FOLFIRINOX (folinic acid, fluorouracil, irinotecan, and oxaliplatin). SMARCA4 knockdown led to a remarkable 86-fold increased sensitivity to oxaliplatin (Figure 1B, 1D).

We next evaluated other chemotherapeutic agents commonly used against pancreatic cancer, including agents that damage DNA by distinct mechanisms (other than DNA crosslinking), as well as agents thought to work predominantly by mechanisms other than damaging DNA. SMARCA4 knockdown resulted in a substantial 29-fold increased sensitivity to irinotecan (Figure 1B, 1E), a DNA topoisomerase I inhibitor that induces DNA single-strand breaks, and also a component of the FOLFIRINOX combination. SMARCA4 knockdown was associated with a more modest, 12-fold increased sensitivity to 5-fluoruracil (Figure 1B, 1F), also part of FOLFIRNOX, and thought to act principally by inhibiting thymidylate synthase (thereby depleting thymidine pools needed for DNA synthesis). Notably, SMARCA4 knockdown had only a minimal effect on gemcitabine sensitivity (1.7-fold IC_50_ reduction) (Figure 1B, 1G). Gemcitabine, a nucleoside analog thought to act predominantly by inhibiting DNA synthesis without inducing DNA breaks [23], has been the mainstay chemotherapy against pancreatic cancer. Likewise, SMARCA4 knockdown only very modestly impacted sensitivity to paclitaxel (4.0-fold IC_50_ reduction) (Figure 1B, 1H), an agent that stabilizes microtubules thereby interfering with mitosis (without damaging DNA).

### SMARCA4 knockdown in human pancreatic ductal epithelial cells results in impaired DNA damage response and increased apoptosis

**Figure 2:**
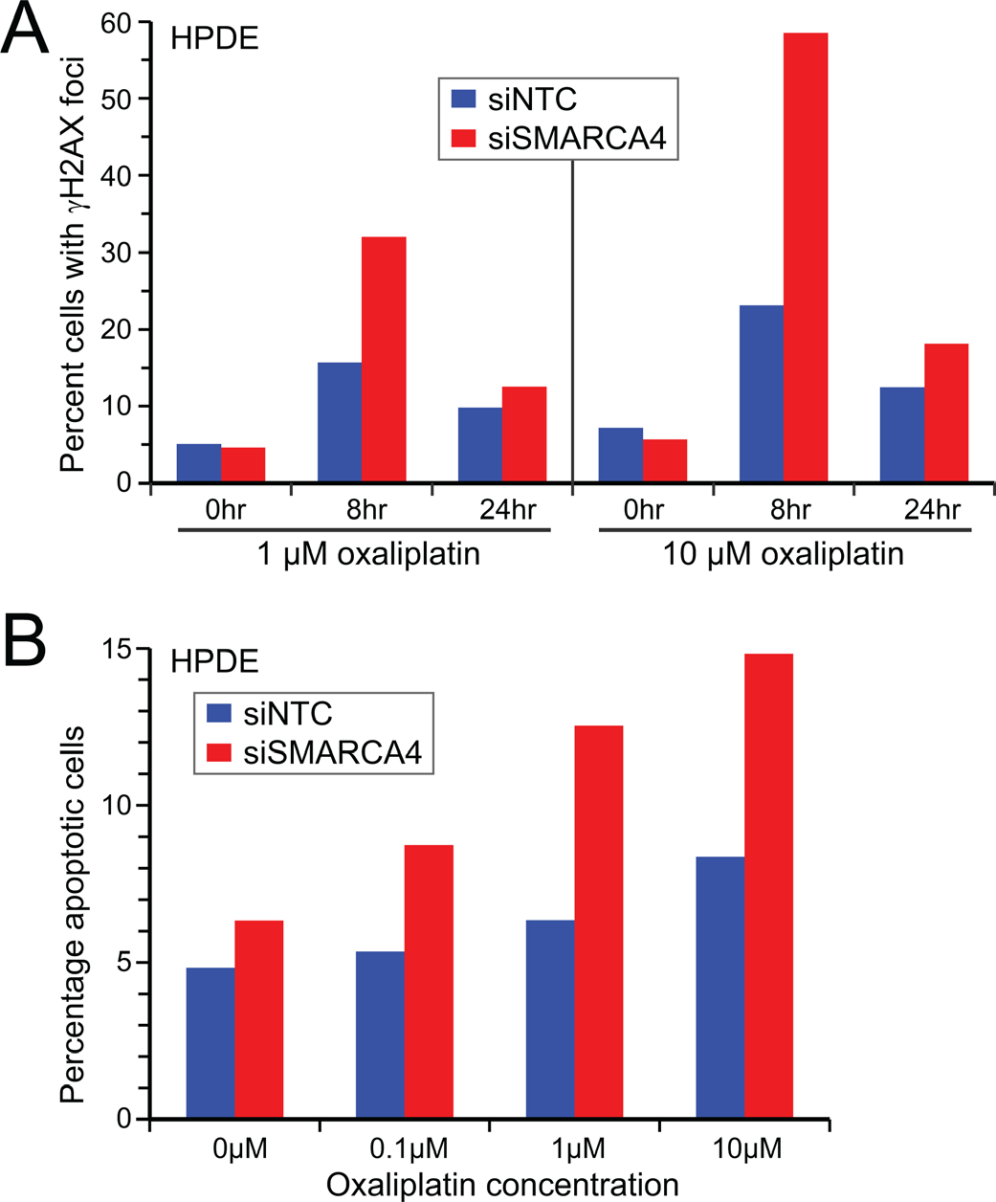
SMARCA4 knockdown in human pancreatic ductal epithelial cells results in impaired DNA damage response and increased apoptosis (**A**) Oxaliplatin treatment leads to increased γH2AX levels (8 hrs) and delayed γH2AX clearance (24 hrs) in HPDE cells following SMARCA4 (*vs.* NTC) knockdown. (**B**) Oxaliplatin treatment results in increased apoptosis in HPDE cells following SMARCA4 (*vs.* NTC) knockdown. γH2AX and apoptosis assayed by flow cytometry (> 10,000 cells); representative results shown.

Our finding that SMARCA4 knockdown sensitized normal human pancreatic epithelial cells to chemotherapeutic agents, especially DNA damaging compounds (cisplatin, oxaliplatin and irinotecan), is consistent with SWI/SNF dysfunction compromising effective DNA damage repair. To verify this expectation, we treated SMARCA4 knockdown (*vs*. control) HPDE cells with oxaliplatin, and then monitored DNA damage response/repair as appearance and subsequent clearance of γH2AX. Oxaliplatin introduces intra- and inter-strand crosslinks into DNA, leading to double-strand breaks repaired through the homologous recombination and Fanconi repair pathways [24], where induction and recruitment of γH2AX is an early step. In response to oxaliplatin (1 µM and 10 µM), HPDE cells with SMARCA4 knockdown showed increased γH2AX levels at 8 hrs and 24 hrs post treatment (i.e. delayed clearance) (Figure 2A), indicative of impaired DNA damage response/repair. Consistent with this finding, HPDE cells with SMARCA4 knockdown (*v*s. control cells), challenged with oxaliplatin, showed increased cell death by apoptosis (Figure 2B).

### SMARCA4 restoration in SMARCA4-deficient pancreatic cancer cells reduces sensitivity to DNA-damaging agents

The above experiments indicated that SWI/SNF dysfunction (by SMARCA4 knockdown) sensitizes pancreatic epithelial cells to chemotherapy agents, and principally those that induce DNA breaks (cisplatin, oxaliplatin, irinotecan). To independently confirm these findings, we carried out the converse set of experiments, namely restoring SMARCA4 expression in SMARCA4-deficient pancreatic cancer cells, and evaluating altered chemo sensitivities.

**Figure 3:**
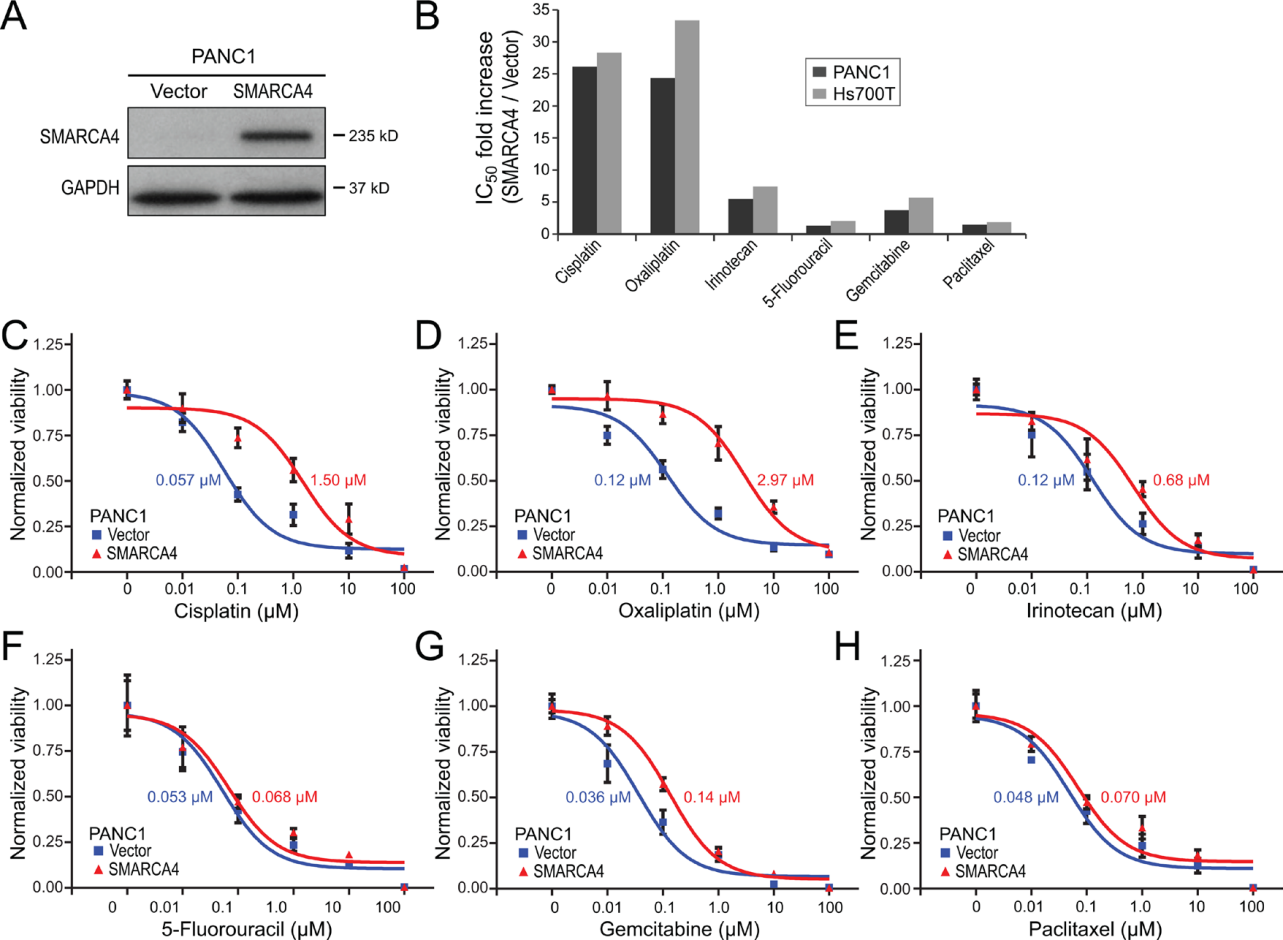
SMARCA4 re-expression in SMARCA4-deficient pancreatic cancer cells reduces sensitivity to DNA-damaging agents (**A**) SMARCA4 re-expression in SMARCA4-defieicint PANC1 cells confirmed by western blot, compared to empty vector control; GAPDH serves as a loading control. (**B**) Bar graph summarizing fold-increases in IC_50_ with SMARCA4 (*vs.* empty vector) re-expression, in PANC1 and Hs700T cells, for 6 different chemotherapy agents. (**C**–**H**) Dose-response curves comparing PANC1 cell viability with SMARCA4 (*vs.* empty vector) expression, for (C) cisplatin, (D) oxaliplatin, (E) irinotecan, (F) 5-fluoruracil, (G) gemcitabine, and (H) paclitaxel. IC_50_ values indicated.

The human pancreatic cancer cell line PANC1 is deficient in SMARCA4 protein, in part from a genomic rearrangement at that locus [7]. Retroviral-based restoration of SMARCA4 expression in PANC1 cells (Figure 3A), compared to empty vector control, led to a respective 26-fold and 24-fold *decreased* sensitivity to cisplatin and oxaliplatin (Figure 3B–3D). Thus, the SMARCA4 deficiency in PANC1 cells, like the SMARCA4 knockdown in HPDE cells, is associated with increased sensitivity to DNA crosslinking agents. SMARCA4 re-expression in PANC1 cells also led to a more modest 5.4-fold reduced sensitivity to the DNA damaging agent irinotecan (Figure 3B, 3E), and to smaller or nominal decreased sensitivities to 5-fluoruracil (4.0 fold), gemcitabine (3.7-fold), and paclitaxel (1.4-fold) (Figure 3B, 3F–3H). Restoration of SMARCA4 expression in a different SMARCA4-deficient pancreatic cancer cell line, Hs700T [7], led to very similar findings, with the most substantial impact being reduced sensitivity to the DNA-damaging agents cisplatin (28-fold), oxaliplatin (33-fold), and irinotecan (7.4-fold) (summarized in Figure 3B).

### Knockdown of other SWI/SNF subunits also sensitizes pancreatic ductal epithelial cells to oxaliplatin

**Figure 4:**
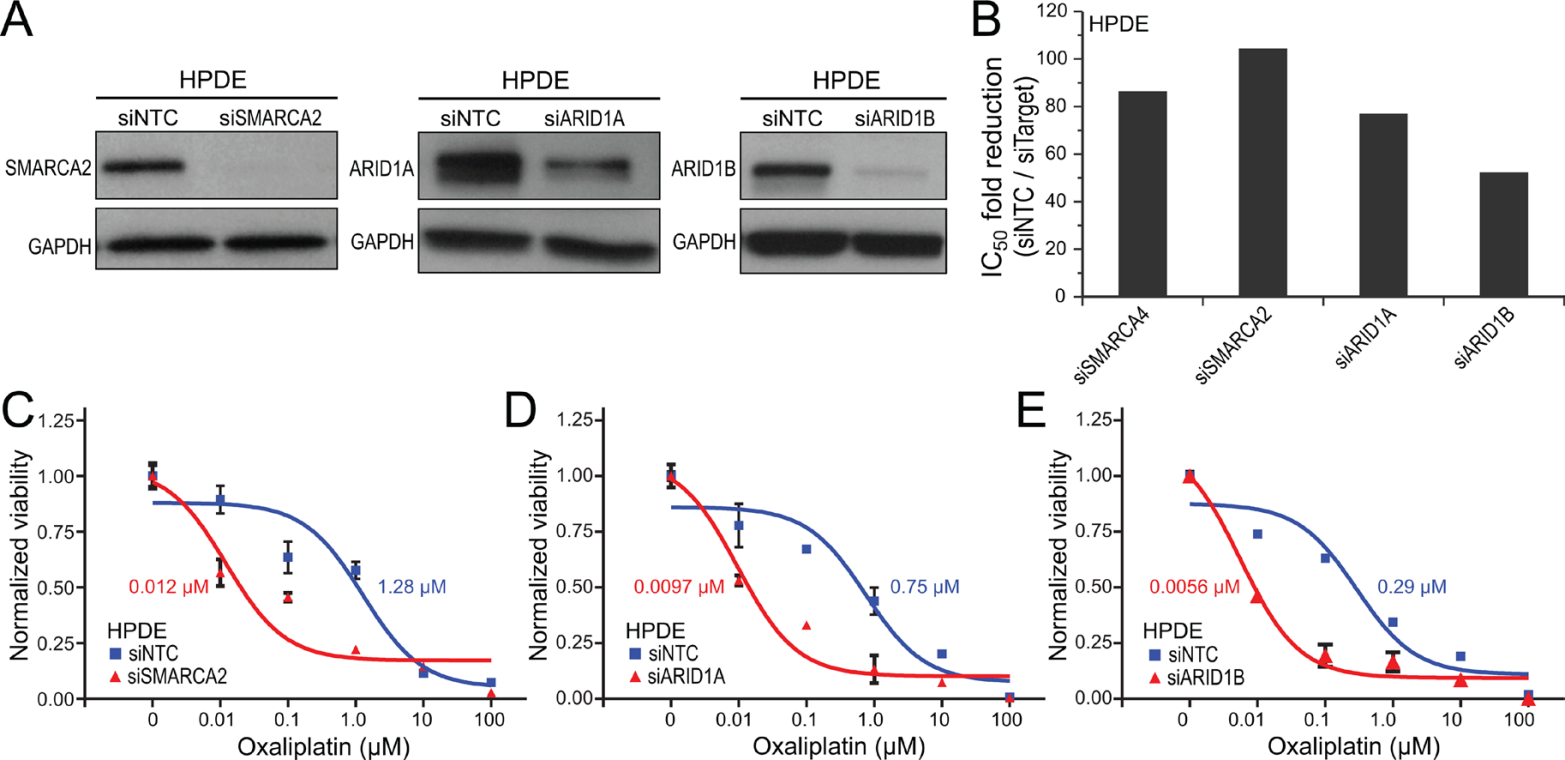
Knockdown of any of several key SWI/SNF subunits sensitizes human pancreatic ductal epithelial cells to oxaliplatin (**A**) siRNA knockdown of SMARCA2, ARID1A and ARID1B in HPDE cells, confirmed by western blot, compared to non-targeting control (NTC); GAPDH serves as a loading control. (**B**) Bar graph summarizing fold-reductions in IC_50_ with knockdown of SMARCA4 (from Figure 1D), SMARCA2, ARID1A, and ARID1B (*vs.* NTC), for oxaliplatin. (**C**–**E**) Dose-response curves comparing HPDE cell viability following knockdown of (C) SMARCA2, (D) ARID1A, and (E) ARID1B (*vs.* NTC), for oxaliplatin. IC_50_ values indicated.

The above knockdown and re-expression studies demonstrated that SWI/SNF dysfunction through SMARCA4 deficiency sensitizes pancreatic epithelial cells to DNA damaging compounds, in particular DNA crosslinking agents. To determine whether the same might extend to other SWI/SNF subunits that we observed to be commonly mutated or deleted in pancreatic cancers, we used RNAi to knockdown SMARCA2 (the alternative enzymatic subunit), ARID1A or ARID1B (DNA targeting/specificity subunits) in normal HPDE cells. Transfection of separate ON-TARGETplus siRNA pools against each of those subunits, compared to non-targeting siRNA control, led to effective target protein knockdown (Figure 4A). Knockdown of SMARCA2, ARID1A, or ARID1B in HPDE cells resulted in a respective 104-fold, 77-fold and 52-fold increased sensitivity to oxaliplatin (Figure 4B–4E), comparable to our previous finding for SMARCA4 knockdown (86-fold).

### Human pancreatic cancers with SWI/SNF aberrations show increased DNA copy number transitions and responsiveness to platinum-based therapy

Our cell culture experiments showed that impairing SWI/SNF function by depletion of any one of several key subunits appears sufficient to compromise DNA damage repair and sensitize pancreatic epithelial cells to DNA crosslinking agents. These findings suggest that patients with pancreatic cancers harboring SWI/SNF aberrations might find particular benefit from DNA crosslinking agents. To extend our cell culture findings to patient samples, we made use of genomic data generated from The Cancer Genome Atlas (TCGA). The latest TCGA build included 185 pancreatic ductal adenocarcinoma cases, profiled for gene mutations (whole exome sequencing), DNA copy number alterations (Affymetrix SNP arrays), and gene expression (RNAseq), with detailed clinical annotations including treatment histories and clinical outcomes available for a subset of 115 cases.

For our analysis, we functionally defined SWI/SNF aberrant cancers as those harboring an aberration in any of the five subunits found commonly-mutated in pancreatic cancer (*SMARCA2*, *SMARCA4*, *ARID1A*, *ARID1B*, *PBRM1*) [7], where aberrations comprised any of (i) Somatic mutations (non-silent SNVs, frameshifting indels, stop-gains, or splice-site) called from whole-exome sequencing data; (ii) Intragenic DNA rearrangements (copy number transitions) called from Affymetrix SNP Array data; (iii) Deep deletions (log_2_ copy number ratio ≤-0.5) quantified from Affymetrix SNP Array data; and/or (iv) Markedly reduced expression (bottom 10th percentile) scored from Illumina RNAseq data. We excluded 3 cases with known pathogenic mutations in *BRCA1*, *BRCA2* or *ATM*, since those “BRCAness” genes have previously been associated with platinum sensitivity [25], and could therefore confound the analysis.

**Figure 5:**
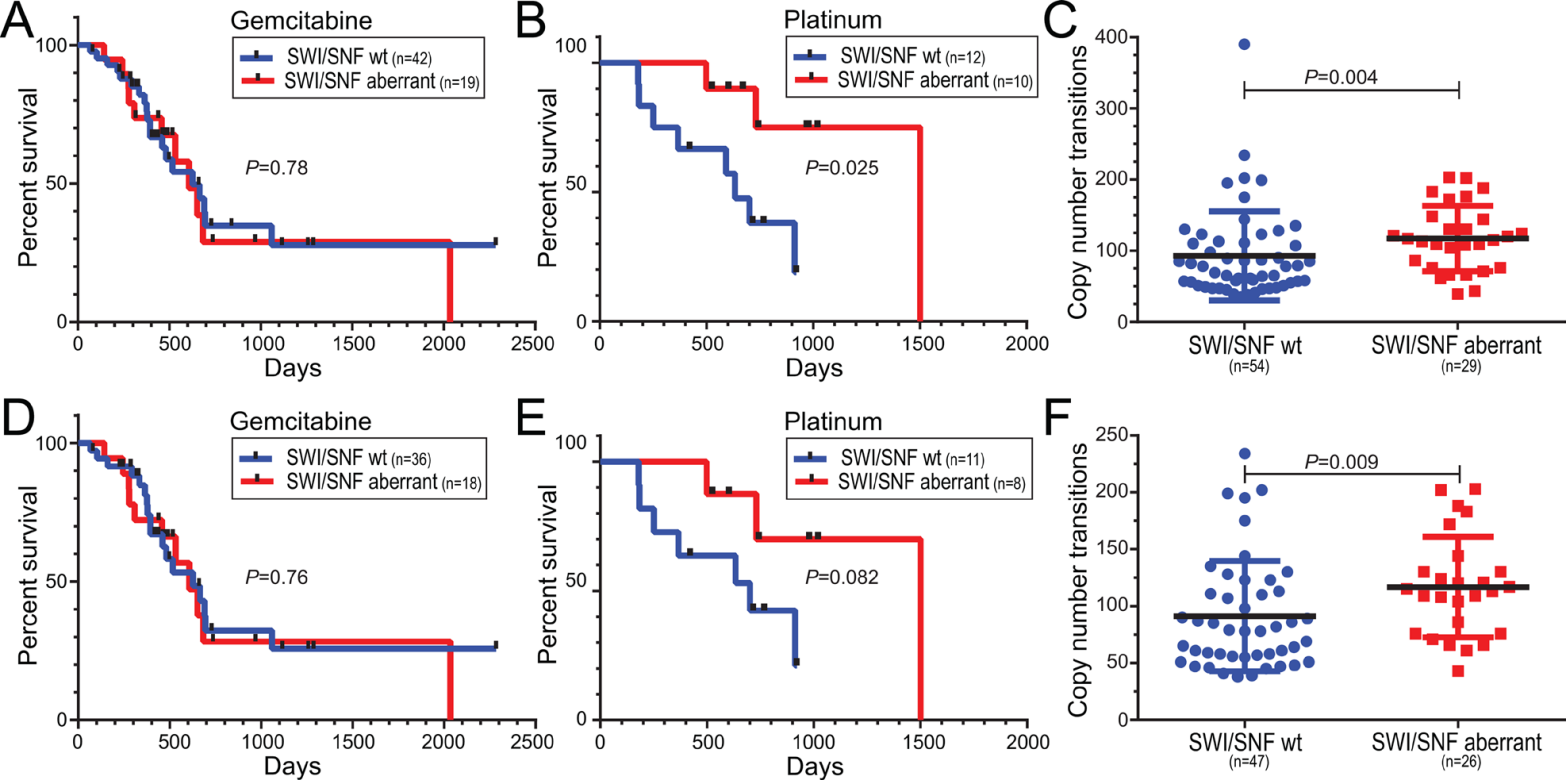
Human pancreatic ductal adenocarcinomas with SWI/SNF aberrations exhibit responsiveness to platinum-based treatment regimens and increased DNA copy number transitions (**A**, **B**) Pancreatic cancer cases with SWI/SNF aberrations are associated with improved overall survival in patients receiving (B) platinum-based therapy, but not (A) gemcitabine alone. Kaplan-Meier curves with log-rank *P*-values shown. (**C**) Pancreatic cancers with SWI/SNF aberrations harbor significantly more DNA copy number transitions (segments). Scatter plots show mean with SD, along with Mann-Whitney *P*-values. (**D**–**F**) Same analyses as above, but omitting samples flagged by the Expert Pathology Committee (EPC) (see comments in main text).

Among 61 pancreatic cancer patients treated with gemcitabine as single-agent therapy, we found no difference in overall survival between pancreatic cancers with and without SWI/SNF aberrations (Figure 5A). Remarkably, however, among 22 patients treated with a platinum agent (cisplatin or oxaliplatin), either alone or as part of a combination regimen (e.g. FOLFIRINOX), those pancreatic cancers with SWI/SNF aberrations were associated with significantly improved overall survival (*P* = 0.037, log-rank test) (Figure 5B). Notably, the pancreatic cancers with SWI/SNF aberrations also harbored significantly more genomic copy number alterations (*P* = 0.004, Mann-Whitney *U*-test) (Figure 5C), consistent with impaired DNA double-strand break repair. Ten of the 83 cases had been flagged by the TCGA Expert Pathology Committee (EPC), where H&E sections apposing the profiled tissue were annotated mainly as atrophic pancreas (Supplementary Table 1). While by genomic analysis those specimens displayed copy number profiles consistent with pancreatic cancer (Supplementary Figure 1), the same trends in survival and copy number transitions held even when those samples were omitted from the analysis (Figure 5D–5F).

## DISCUSSION

In our studies, we found that SWI/SNF dysfunction by engineered subunit depletion in normal human pancreatic epithelial cells, or by somatic mutation in human pancreatic cancer cells, sensitizes those cells to DNA damaging agents, and in particular the DNA crosslinking agents cisplatin and oxaliplatin. Consistent with this finding, patients with pancreatic cancers harboring SWI/SNF aberrations exhibited increased responsiveness (measured by overall survival) to DNA crosslinking agents, but not to the non-DNA damaging (break inducing) compound gemcitabine.

SWI/SNF dysfunction has been reported to contribute to cancer through varied mechanisms [10], but most studies have focused on its role in transcriptional regulation. Here, we found that SWI/SNF dysfunction (by SMARCA4 depletion) in pancreatic epithelial cells impairs DNA damage response/repair, consistent with prior reports in other cell types [17–19]. Specifically, we found an impaired response to a DNA crosslinking agent (oxaliplatin) that generates DNA double-strand breaks. Consistent with this, we found a modest but significant increase in DNA copy number transitions, a consequence of DNA double-strand breaks, in human pancreatic cancers harboring SWI/SNF aberrations. Whether SWI/SNF aberrations might promote genetic instability [26], enabling cancer development or progression, will require further investigation.

Platinum-based therapies have shown promise in pancreatic cancer, particularly in combinations such as FOLFIRINOX, but also exhibit increased toxicity [4, 27]. Importantly, our studies suggest that SWI/SNF functional status might provide a “theragnostic” biomarker to identify those patients most likely to benefit from platinum therapies. Though the survival gain was modest, extending survival even weeks or months in patients with pancreatic cancer would have substantial impact.

We note several limitations of our study, including that the number of patients was relatively small, and that the patients were not managed within a uniform treatment protocol (albeit standard therapy regimens were followed). Furthermore, we defined SWI/SNF dysfunction by genomic and transcriptomic data (mutations, rearrangements, deletions, diminished expression) for select subunits. Nonetheless, given these limitations it is remarkable that we observed an association between SWI/SNF status and platinum sensitivity. Our findings provide strong rationale for follow-up investigations, using larger cohorts, ideally as part of retrospective or prospective clinical trials. Future studies should also address the optimal assay for SWI/SNF dysfunction, which might include immunohistochemistry on select subunits, or some combination of DNA, RNA and protein analysis.

SWI/SNF mutations have now been reported in many other cancer types, with the highest frequencies found in ovarian (clear cell type), renal, hepatocellular, and gastric cancers [12, 13]. That some cancer types exhibit clear predilections for specific mutated subunits (like *ARID1A* in ovarian clear cell carcinoma) suggests that SWI/SNF has distinct functions in different normal tissues and derived cancers. As such, it will be important to investigate whether SWI/SNF dysfunction in other cancer types might also predict clinical response to DNA crosslinking agents.

DNA crosslinks and the resultant DNA double-strand breaks are repaired through the homologous recombination and Fanconi repair pathways [24], where BRCA1 and associated proteins play a key role. Cancers harboring mutations in “BRCAness” genes [25], including *BRCA1*, *BRCA2*, *ATM* and *PALB2*, have been found to be sensitive to DNA crosslinking agents. Notably, such tumors are also sensitive to Poly ADP ribose polymerase (PARP) inhibition, presumably because in the absence of functional PARP, replication forks stalled at DNA single-strand breaks (normally repaired by base excision repair utilizing PARP) collapse to form double-strand breaks which rely on BRCA proteins for repair. BRCA impairment together (in synthesis with) with PARP inhibition creates a “synthetic lethality” [28]. Of note, it was recently reported that SWI/SNF interacts with BRCA1 to effect DNA repair (and mammary cell differentiation) [29]. It will therefore be of interest to determine whether SWI/SNF dysfunction also sensitizes pancreatic cancer cells to PARP inhibition, either alone or in combination with DNA damaging agents. In this regard, it was recently reported that SWI/SNF dysfunction sensitizes HeLa (D98) cervical cancer cells to single-agent PARP inhibition (veliparib) [30].

Several recent studies have reported promising strategies to therapeutically exploit SWI/SNF dysfunction in human cancer [15]. SWI/SNF dysfunction has been reported to lead to lost antagonism of Polycomb Repressive complexes in rhabdoid tumors, and such cancers have been found sensitive to inactivation of EZH2 (the catalytic subunit of PRC2) [31, 32]. In addition, ovarian and lung cancers deficient in one SWI/SNF subunit appear sensitive to loss (by RNAi depletion) of the alternative subunit [33, 34], e.g. SMARCA2 depletion in SMARCA4-deficient lung cancer cells. Our finding that SWI/SNF dysfunction sensitizes pancreatic cancer cells to DNA crosslinking agents provides a new and orthogonal (potentially combinable) avenue to therapeutically target SWI/SNF dysfunction.

In summary, we find that SWI/SNF dysfunction sensitizes human pancreatic cancer cells to DNA damaging agents, in particular DNA crosslinking agents, and that SWI/SNF functional status may provide a useful biomarker to predict which patients are likely to benefit from platinum-containing chemotherapy regimens.

## MATERIALS AND METHODS

### Cell lines

Human pancreatic ductal epithelial (HPDE) cells were obtained from the originator (Dr. Ming Tsao; University of Toronto) [21], and grown in keratinocyte serum-free media (Invitrogen). PANC1 and Hs700T human pancreatic cancer cells were obtained from ATCC, and grown in DMEM media supplemented with 10% FBS. All cell lines were used directly (or from early freezes made) within 6 months of receipt.

### Knockdown and re-expression studies

RNAi knockdown of SMARCA4, SMARCA2, ARID1A and ARID1B was done by Lipofectamine 2000 (Invitrogen) transfection of Dharmacon ON-TARGETplus siRNA pools (20nM final), in comparison to Non-Targeting Control (NTC) siRNA pool. Previous studies demonstrated on-target knockdown, as two or more individual siRNAs from each pool exhibited similar knockdown efficiency and cellular phenotype [7]. Re-expression of SMARCA4 was done by retroviral transduction (pBABE-puro-SMARCA4) [7], in comparison to empty vector control. Knockdown and/or re-expression were confirmed by western blotting of whole cell lysates, using the following primary antibodies: SMARCA4 (sc-17796; Santa Cruz), SMARCA2 (sc-166579; Santa Cruz), ARID1A (ab176395; Abcam), ARID1B (ab57461; Abcam), and GAPDH (sc-25778; Santa Cruz). Knockdown efficiency was quantified using ImageJ.

### Cell viability, γH2AX levels, and apoptosis assays

Cisplatin, oxaliplatin, irinotecan, 5-fluoruracil, gemcitabine and paclitaxel were purchased from Sigma, and used at the concentrations indicated. For knockdown studies, 50K cells were seeded per 6-well plate well, transfected with siRNA the following day, and then fresh media with chemotherapeutic agent (or vehicle control) added one day later. For re-expression studies, 50K cells were seeded per 6-well plate well, and then fresh media with chemotherapeutic agent added one day later. Cell viability was measured by WST-1 assay (Roche) following 72 hrs treatment, and IC_50_ values determined by fitting a sigmoidal curve (GraphPad Prism). All assays were done in biological triplicate. γH2AX levels (a surrogate for γH2AX foci) were quantified by flow cytometry (> 10,000 cells) using a FITC-conjugated anti-phospho-H2A.X(Ser139) antibody (H2A.X Phosphorylation Assay Kit; EMD Millipore). Apoptosis was measured by flow cytometry (> 10,000 cells) by dual YO-PRO-1 and PI staining (Membrane Permeability/Dead Cell Apoptosis Kit; Invitrogen).

### Analysis of TCGA data

TCGA pancreatic adenocarcinoma (PAAD) genomic data (processed) were downloaded from Broad GDAC Firehose, along with the most recent clinicopathologic annotations from TCGA Data Portal and NCI Genomic Data Commons (last accessed March 4, 2017). Cases with SWI/SNF aberration were determined as aberrations affecting any of the five commonly-mutated SWI/SNF subunits (*SMARCA2*, *SMARCA4*, *ARID1A*, *ARID1B*, *PBRM1*) [7], where aberrations comprised any of (i) Somatic mutations (non-silent SNVs, frameshifting indels, stop-gains, or splice-site) from whole-exome sequencing data (Mutation Packager Oncotated calls); (ii) Intragenic DNA rearrangements (copy number transitions) from Affymetrix SNP Array 6.0 data (Segmented SCNA minus germline CNV calls); (iii) Deep deletions (log_2_ copy number ratio ≤−0.5) from Affymetrix SNP Array 6.0 data (Segmented SCNA minus germline CNV calls); and/or (iv) Markedly reduced expression (bottom 10^th^ percentile) from Illumina RNAseq data (RNAseqV2 RSEM genes normalized). Total DNA copy number transitions were defined by the sum of called segments from Affymetrix SNP Array 6.0 data (Segmented SCNA minus germline CNV calls). Survival analysis was done by the Kaplan-Meier method, using overall survival and log-rank test. Where indicated, samples harboring a pathogenic mutation (ClinVar annotation [35]) in pancreatic cancer associated “BRCAness” genes [25] (*BRCA1*, *BRCA2*, *ATM*, *PALB2*), and/or flagged by the Expert Pathology Committee (EPC), were omitted from analysis. TCGA specimen annotations are summarized in Supplementary Table 1.

## SUPPLEMENTARY MATERIALS FIGURE AND TABLE





## Abbreviations

CNV: Copy number variant
EPC: Expert Pathology Committee
FOLFIRINOX: Folinic acid, fluorouracil, irinotecan, and oxaliplatin
HPDE: Human pancreatic ductal epithelial cells
NTC: Non-targeting control
PAAD: Pancreatic adenocarinoma
siRNA: Small interfering RNA
SNV: Single nucleotide variant
SWI/SNF: SWItch/Sucrose NonFermentable
TCGA: The Cancer Genome Atlas
