# Supplementary material for: SWI/SNF aberrations sensitize pancreatic cancer cells to DNA crosslinking agents
Source: Oncotarget. 2017 Aug 8;9(11):9608–17. doi: 10.18632/oncotarget.20033 (PMC5839388; doi:10.18632/oncotarget.20033)
Supplement: Supplementary file 1 [file oncotarget-09-9608-s001.pdf]

SWI/SNF aberrations sensitize pancreatic cancer cells to DNA crosslinking agents

SUPPLEMENTARY MATERIALS

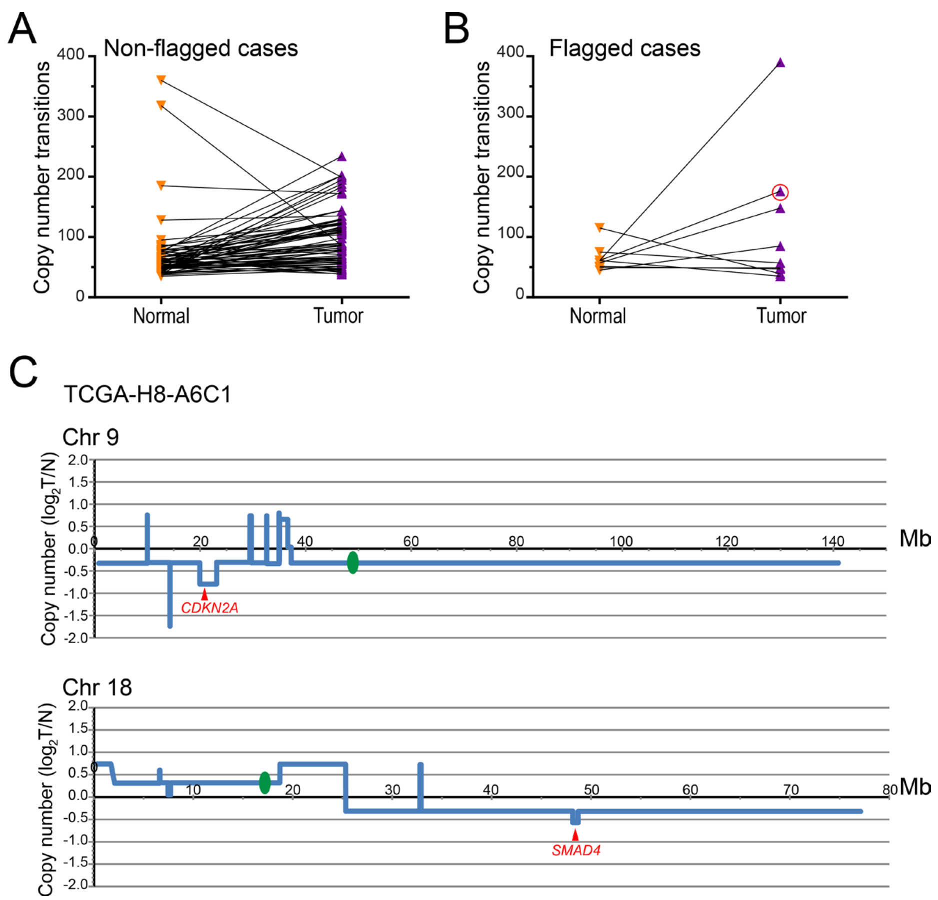

Supplementary Figure 1: Attributes of TCGA cases non-flagged and flagged by the Expert Pathology Committee. (**A**, **B**) Number of DNA copy number transitions called in matched normal (orange triangle) and pancreatic cancer (purple triangle) specimens, from cases (A) not flagged, or (B) flagged by the EPC. Most flagged samples were called atrophic pancreas by H&E analysis of the apposing tissue face. Note, for both non-flagged and flagged cases, most tumors exhibit more copy number transitions compared to their matched normal. Copy number transitions in normal samples may reflect personal CNVs or TCGA miscalls. Case TCGA-H8-A6C1 is highlighted by red circle. (**C**) Flagged cases include copy number profiles consistent with pancreatic cancer, shown here for TCGA-H8-A6C1 (apposing H&E section annotated as "atrophic pancreas"). Log_2 Tumor/Normal copy number profiles drawn (blue lines) for chromosomes 9 and 18; centromeres marked by green ellipse. Note the focal deletions of pancreatic cancer tumor suppressor genes *CDKN2A* (chr 9p) and *SMAD4* (chr 18q).

Supplementary Table 1: Clinicopathologic features of TCGA cohort. See_Supplementary_Table 1
